# Repurposed Drugs in Gastric Cancer

**DOI:** 10.3390/molecules28010319

**Published:** 2022-12-30

**Authors:** Diana Araújo, Eduarda Ribeiro, Irina Amorim, Nuno Vale

**Affiliations:** 1 OncoPharma Research Group, Center for Health Technology and Services Research (CINTESIS), Rua Doutor Plácido da Costa, 4200-450 Porto, Portugal; 2 Institute of Biomedical Sciences Abel Salazar (ICBAS), Universidade do Porto (UP), Rua de Jorge Viterbo Ferreira 228, 4050-313 Porto, Portugal; 3 Institute for Research and Innovation in Health (i3S), Universidade do Porto, Rua Alfredo Allen, 208, 4200-135 Porto, Portugal; 4 Institute of Molecular Pathology and Immunology, University of Porto (IPATIMUP), Rua Júlio Amaral de Carvalho, 45, 4200-135 Porto, Portugal; 5 CINTESIS@RISE, Faculty of Medicine, University of Porto, Alameda Professor Hernâni Monteiro, 4200-319 Porto, Portugal; 6 Department of Community Medicine, Health Information and Decision (MEDCIDS), Faculty of Medicine, University of Porto, Rua Doutor Plácido da Costa, 4200-450 Porto, Portugal

**Keywords:** gastric cancer, anticancer drugs, repurposing drugs

## Abstract

Gastric cancer (GC) is one of the major causes of death worldwide, ranking as the fifth most incident cancer in 2020 and the fourth leading cause of cancer mortality. The majority of GC patients are in an advanced stage at the time of diagnosis, presenting a poor prognosis and outcome. Current GC treatment approaches involve endoscopic detection, gastrectomy and chemotherapy or chemoradiotherapy in an adjuvant or neoadjuvant setting. Drug development approaches demand extreme effort to identify molecular mechanisms of action of new drug candidates. Drug repurposing is based on the research of new therapeutic indications of drugs approved for other pathologies. In this review, we explore GC and the different drugs repurposed for this disease.

## 1. Introduction

Gastric cancer (GC) is one of the major causes of death worldwide [1,2], ranking as the fifth most incident cancer in 2020 and the fourth leading cause of cancer mortality [3]. The high mortality rate is associated with the lack of clinical signs in the early stages and insufficient screening programs and health care assistance in developing countries. Thus, the majority of GC patients are in an advanced stage at the time of diagnosis, presenting a poor prognosis [4]. GC symptoms and clinical signs are vague and nonspecific, and mild to absent during the early stages of the disease, with the most common being loss of appetite and dysphagia, abdominal discomfort and dyspepsia, weight loss, weakness due to anaemia, nausea and vomiting and melena [1,2,5,6]. The duration of the symptoms can range from 3 months to more than 1 year. However, neither these symptoms nor routine physical exams can provide an early diagnosis of this disease. The diagnosis is usually established by upper gastrointestinal endoscopy, followed by visualization and biopsy to confirm the diagnosis [5].

The most significant prognostic factors of GC at the time of diagnosis are the depth of invasion and the presence of regional and distant metastasis [2,5], with local involvement of the duodenum, pancreas and retroperitoneum frequently observed [2]. The survival rate decreases as the tumour extension and the number of lymph nodes affected increases. The prognosis is usually poorer in high-grade and diffuse-type carcinomas [5]. Current GC treatment approaches involve endoscopic detection, gastrectomy and chemotherapy or chemoradiotherapy in an adjuvant (treatment administration after a potentially curative resection) [1] or neoadjuvant setting (therapy before potentially curative resection) [1,7]. In metastatic cases, chemotherapy remains the most important therapy and palliative care [2,5,8]. Despite progress in treatment efficacy, the outcome is still poor. To overcome these issues, targeted therapies and immunotherapies with immune checkpoint inhibitors are being studied [5]. In this review, we explore GC and the different drugs repurposed for this often-fatal disease.

## 2. Epidemiology and Risk Factors

GC’s incidence varies according to gender and geographical variability. Men are two-fold more susceptible than women. GC is the most frequently diagnosed cancer and the leading cause of cancer-related death in men from South-Central Asian countries. The incidence rates are the highest in Eastern Asia and Eastern Europe, while in Northern America and Northern Europe, they are usually low, being similar to those in Africa [9].

Different factors have been associated with GC incidence, such as diet, smoking, alcohol consumption and family history [2,5,10]. It is known that fruit and vegetables are protectors against GC development; on the other hand, smoked or salted foods may enhance GC progression [10]. A study showed that smoking and alcohol consumption may be independent risk factors for GC development in high-risk populations [11].

Some pathological conditions can also be a precursor of this disease, such as pernicious anaemia, achlorhydria atrophic gastritis, gastric ulcers and adenomatous polyps [2,5]. The development of GC has also been associated with chronic infection with *Helicobacter pylori* [2,5]. This infection can cause multifocal atrophic gastritis, which is associated with mucosal atrophy, a reduction in parietal cells and acid secretion, intestinal metaplasia and therefore an increased risk of gastric adenocarcinoma development [2]. Parsonnet et al. have shown that there is a three-fold-increased risk of GC in individuals with H. pylori infection [12]. However, the association between *H. pylori* infection and gastric carcinogenesis is still debated, since some mechanisms are not yet completely understood. Nevertheless, *H. pylori* may play an important role in epithelial-to-mesenchymal transition (EMT) induction within gastric mucosa, which depends on the presence of specific *H. pylori* virulence factors. Therefore, exploring the virulence factors as potential molecular targets may allow new therapeutic approaches for GC treatment [13].

**Figure 1:**
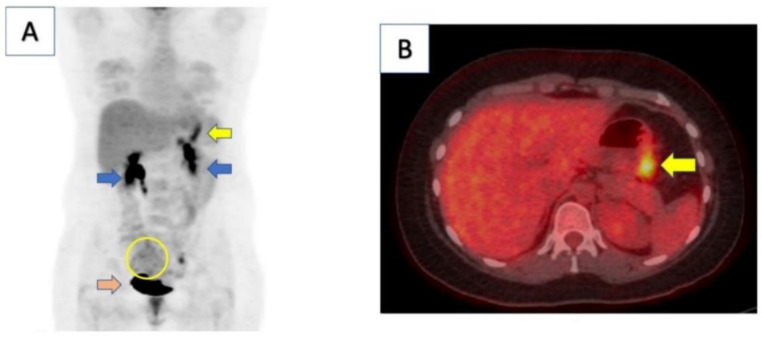
Computed tomography (CT)/positron emission tomography (PET) scan of patient demonstrated moderate focal uptake along gastric body consistent with known malignancy (yellow arrow in panel (**A**,**B**)). There was also a large, mildly hypermetabolic right adnexal area with heterogenous uptake, indicating metastatic involvement (yellow circle). Kidneys (blue arrows) and bladder (orange arrow) demonstrate physiologic uptake. Reproduced from [15].

In sum, host and environmental factors can determine the development of GC [2,5,14]. The patient had previously been treated for H. pylori, which is the main risk factor for developing gastric cancer (Figure 1). The image illustrates that Helicobacter is one of the possible causes of gastric cancer, but it is not the only one and does not represent the totality of the others.

## 3. Classification Subtypes

Adenocarcinoma is the most common gastric malignant neoplasia, corresponding to more than 90% of all gastric malignancies [1,2]. Adenocarcinomas are categorized based on their location and morphology and frequently involve the gastric antrum and the lesser curvature. Regarding the histology, the intestinal type tends to form bulky masses and present glandular structures, forming an exophytic mass or an ulcerated tumour; the diffuse type diffusely infiltrates the wall and thickens it, being composed of discohesive cells such as signet ring cells [1,2,14]. These subtypes present different clinical features, outcomes and pathogeneses, since the intestinal type prevails in high-risk areas with a male-to-female ratio of 2:1 and appears at a mean age of 55 years, and its development is related to precursors lesions, since it progresses from normal gastric epithelium to chronic atrophic gastritis to intestinal metaplasia, then to dysplasia and cancer, being associated with *H. pylori* infection, while the incidence of the diffuse type is similar worldwide, with no precursor factors identified and no differences between genders. It is more aggressive and has a higher potential to invade, thus presenting a poorer outcome and being characterized by loss of expression of E-cadherin (mutation in the *CDH1* gene) [1,2,14]. The germline loss-of-function mutations in the tumour suppressor gene *CDH1*, encoding the cell-to-cell adhesion protein E-cadherin, is associated with familial GC [1,2]. This mutation can be found in about 50% of sporadic diffuse GC. However, in the remaining GC cases, there is a decrease in E-cadherin expression due to hypermethylation and silencing of the *CDH1* promoter. In addition, mutations in the *CDH1* gene can also be found in breast sporadic and familial lobular carcinoma. Additionally, mutation of TP53 can be found in most sporadic GCs of both diffuse and intestinal types. [2]. However, the outcome depends on the tumour, node and metastasis (TNM) stage [1].

The molecular classification of gastric cancer is extremely important in prognosis, treatment and staging [1]. The Cancer Genome Atlas (TCGA) described four subtypes of GC based on a molecular classification: Epstein–Barr-virus-associated; microsatellite instability; genomically stable; and chromosomal instability. It is known that genomically stable tumours correspond to the diffuse type and those that display chromosomal instability are the intestinal type. These molecular characteristics taken together with histopathology are particularly valuable for a patient’s staging and treatment [16]. Furthermore, the molecular subtypes enable the use of targeted therapy [1,16].

## 4. Treatment Strategies

### 4.1. Surgical Resection Approach

Surgical resection represents the elective treatment for locally advanced GC and gastric adenocarcinoma [2,5], with a five-year survival rate of 90% in early GC and less than 20% in advanced cases [2]. However, families harbouring *CDH1* gene germline mutations present a 70% lifetime risk of developing GC, with early total gastrectomy being recommended for these patients due to the failure of effective early detection by endoscopy [5]. Nevertheless, most patients relapse even after resection; thus, the development of pre- and postoperative systemic and regional adjuvant therapies is important to overcome those poor results [1]. The early stages of GC are usually treated endoscopically or surgically [17], and patients in the intermediate stages (stages II and III) receive combined modality therapies [6,17]. Stage IV cases are usually incurable, and the therapies consist of palliative care [17].

### 4.2. Perioperative Chemotherapy

In patients with resectable GC, perioperative (pre- and postoperative) chemotherapy with a platinum–fluoropyrimidine combination is recommended [6]. The United Kingdom Medical Research Council Adjuvant Gastric Infusional Chemotherapy (MAGIC) trial assessed the administration of a chemotherapy protocol with three preoperative and three postoperative cycles of epirubicin, cisplatin and infused fluorouracil (ECF) in patients with resectable GC, which demonstrated an improvement in progression-free and overall survival [18]. An increase in the curative resection rate and disease-free and overall survival was demonstrated in the randomized phase III trial by the Fédération Nationale des Centres de Lutte contre le Cancer (FNCLCC) and the Fédération Francophone de Cancérologie Digestive (FFCD), where patients with resectable GC received two or three preoperative cycles of cisplatin and fluorouracil [19]. Another trial assessed the efficacy of two cycles of cisplatin followed by d-L-folinic acid and fluorouracil in patients with locally advanced GC, which improved the resection rate. However, it was not successful in representing a survival benefit [20]. Capecitabine and oxaliplatin were tested as substitutes to fluorouracil and cisplatin, respectively, for untreated advanced esophagogastric cancer and showed that capecitabine and oxaliplatin are as efficient as fluorouracil and cisplatin [21]. Therefore, the use of any fluoropyrimidine–platinum doublet or triplet pre-surgery, being the combination of cisplatin–fluorouracil and epirubicin, for 2–3 months, presented the best treatment option [6].

### 4.3. Adjuvant Therapy

Adjuvant treatments, such as chemoradiotherapy or adjuvant chemotherapy, are advised in patients who have undergone surgery without administration of preoperative chemotherapy [6]. Adjuvant chemotherapy has been proven to be valuable in those previous Asian trials. The Adjuvant Chemotherapy Trial of TS-1 for Gastric Cancer (ACTS-GC) assessed S-1, an oral fluoropyrimidine used in advanced GC, as an adjuvant chemotherapy in patients with curative resectable GC and demonstrated an improvement in the overall survival and relapse-free survival of those patients [22]. The Capecitabine and Oxaliplatin Adjuvant Study in Stomach Cancer (CLASSIC) randomized phase III trial evaluated the role of capecitabine plus oxaliplatin adjuvant after surgery and showed an enhancement in overall and disease-free survival [23,24]. However, in Europe, the use of adjuvant chemotherapy in patients with resected GC is still restricted due to the lack of definitive evidence of its benefit and to the perioperative chemotherapy routinely used [6]. Nevertheless, a meta-analysis study of adjuvant chemotherapy in GC showed that adjuvant fluorouracil-based chemotherapy is related to an enhanced overall survival and reduced risk of death in GC patients, and is thus recommended for patients who have not received perioperative therapy [25]. Despite this, perioperative therapy is the preferred option, since adjuvant chemotherapy is less tolerated than neoadjuvant chemotherapy [6].

### 4.4. Chemotherapy: First-Line Treatment

In patients with advanced GC, the administration of doublet or triplet platinum–fluoropyrimidine combinations is recommended [6]. At the time of diagnosis, the majority of patients with GC present an advanced stage of disease, with the tumour being inoperable or recurring within five years after surgery [8,26]. In these cases, chemotherapy as a systemic treatment should be considered, since it has been proven to improve survival and life quality compared with the best supportive care alone [8,27,28]. Chemotherapy is also the main treatment for metastatic or recurrent disease [5,8]. A study assessed different chemotherapy parameters and protocols for advanced GC: chemotherapy compared to best supportive care; combination and single-agent chemotherapy; and different chemotherapy combinations. Wagner et al. demonstrated that chemotherapy improved survival when compared to the best supportive care, as well as combination chemotherapy in comparison to single-agent 5-fluorouracil (5-FU) [8]. Patients can benefit from two- and three-drug combinations, such as irinotecan, docetaxel, oxaliplatin or 5-FU drugs. Combinations containing docetaxel, as a single-agent or two-drug combination (platinum/5-FU), and irinotecan-containing combinations reveal significant survival improvement. The three-drug regimens containing docetaxel present increased response rates and also increased toxicity. Two- and three-drug procedures can improve survival; however, they are also associated with increased toxicity [8]. The two-drug cytotoxic protocols are preferred for patients with advanced GC due to their lower toxicity; the three-drug regimens should be administered to medically fit patients with a good performance status [29]. In protocols where chemotherapy agents were replaced by irinotecan, there was a survival benefit without increased toxicity. Therefore, combinations containing irinotecan/5-FU are a good choice for first-line therapy [8].

Despite this, special attention should be paid to elderly patients that may benefit from oxaliplatin compared to a cisplatin-based protocol [8], due to its lower toxicity [29], and to patients with locally advanced disease or those younger than 65 years that might benefit from a three-drug protocol, such as 5-FU, docetaxel and oxaliplatin, compared to a two-drug combination of 5-FU and oxaliplatin [8].

In sum, oxaliplatin and capecitabine are appropriate substitutes for cisplatin and 5-FU. In upper gastrointestinal tract malignancies, various drugs, such as oxaliplatin, cisplatin, 5-FU, irinotecan, capecitabine, taxanes and anthracyclines, have at least moderate single-agent activity [1].

### 4.5. Chemotherapy: Second-Line Treatment

Most GC patients present various comorbidities and complications that do not allow the safe administration of a second-line therapy [1]. However, for patients with a satisfactory status, the ramucirumab-plus-paclitaxel combination is suggested as a second-line therapy; for those patients that cannot afford an intensive treatment, single-agent ramucirumab or paclitaxel is a more suitable therapy choice [26].

### 4.6. Molecular Targeted Therapy

Trastuzumab is a humanized monoclonal antibody that targets the human epidermal growth factor receptor-2 (HER-2), inhibits downstream signal activation and promotes antibody-dependent cellular toxicity [30].

In HER-2-positive tumours, trastuzumab combined with capecitabine and 5-FU with cisplatin has shown valuable results [8,31,32]. A phase III clinical trial determined the addition of trastuzumab to chemotherapy as the standard of care in the first-line treatment of advanced HER-2-positive GC [29,33].

A study demonstrated that the overall survival of HER-2-positive patients treated with trastuzumab was significantly improved compared to that of HER-2-negative patients, underlining that the trastuzumab therapeutic approach improves prognosis and is appropriate for HER-2-positive patients [31]. Therefore, all patients should be tested for HER-2 status [8].

### 4.7. Immunotherapy

The immune checkpoint blockade is a therapy approach used in various malignancies, and it comprises monoclonal antibodies that suppress programmed cell death protein 1 (PD-1), PD-L1 and cytotoxic T-lymphocyte antigen 4 (CTLA-4) [30].

The mismatch repair (MMR) genes correct errors that occur in DNA replication. Tumours with defects in this repair system display more mutations than those with intact MMR machinery. MMR-deficient cancers are more likely to respond to the PD-1 blockade due to the infiltration of neoantigens and PD-L1-positive T cells in those tumours [30].

Pembrolizumab is a humanized monoclonal antibody that suppresses PD-1 activity through binding to PD-1 receptors on T cells, thus hindering PD-1 ligands (PD-L1 and PD-L2) from binding. The PD-1 blockade is able to induce the antitumour response, since it involves the removal of the physiologic brake on an active immune system [30].

A randomized phase III trial compared pembrolizumab with paclitaxel in the second-line treatment of advanced GC, and it was demonstrated that pembrolizumab did not enhance survival compared with paclitaxel in the second-line setting. Nevertheless, pembrolizumab exhibited an increasing advantage with higher PD-L1 levels and fewer side effects [34]. Another phase III randomized trial demonstrated that in patients with untreated advanced GC, pembrolizumab was not inferior to chemotherapy and had fewer adverse effects. Pembrolizumab or pembrolizumab plus chemotherapy did not improve overall survival or progression-free survival at the endpoints tested [35].

Nivolumab is also a humanized monoclonal antibody that inhibits PD-1 [30]. A phase III clinical trial showed that nivolumab improved survival when compared with the placebo in pretreated patients with advanced GC. However, this study was performed in an Asian population not tested for PD-L1 expression [36]. A study in Western populations assessed nivolumab alone and in combination with ipilimumab, a monoclonal antibody that inhibits CTLA-4, and demonstrated that it displays antitumour action and long-term overall survival, with a safety profile in patients with chemotherapy-refractory gastroesophageal cancers [37].

Immunotherapy has also been proven to be effective when added to HER-2 therapy. A phase II trial showed that pembrolizumab could be safely used in combination with trastuzumab plus chemotherapy in HER-2-positive metastatic gastroesophageal adenocarcinomas [38].

## 5. Repurposing Drugs

Drug development approaches demand extreme effort to identify molecular mechanisms of action of new drug candidates [39]. In recent years, drug repurposing or repositioning has become an important part of the drug development procedure [40]. Drug repurposing is based on the research of new therapeutic indications of drugs approved for other pathologies [41]. In fact, repurposed drugs have already been through preclinical and some early human clinical trials and in most cases are considered safe with well-known pharmacokinetic profiles. Nevertheless, these drugs have a greater potential of being safe in new conditions and in distinct patient populations [40,41]. Drug repurposing decreases the risks and costs of drug use, and saves time. Therefore, repurposing is beneficial when compared to other drug development approaches [41]. PubMed, MEDLINE, SCOPUS and Google Scholar were searched for relevant studies published up to August 2022 using suitable keywords such as “repurposed drugs gastric cancer” and “gastric cell line drug repurposing”. Several studies have assessed the in vitro and in vivo efficacy of different repurposed drugs to treat GC (Table 1).

### 5.1. Repurposed Drugs in Gastric Cancer

#### 5.1.1. Antidepressant Agents

Fluoxetine is a selective serotonin reuptake inhibitor (SSRI) that suppresses serotonin transporters (SERTs). This drug is commonly used to treat depression, obsessive compulsive disorder, panic and anxiety disorders, post-traumatic stress disorder, fibromyalgia and neuropathic pain [42,43,44,45,46]. Depression may arise in patients with cancer, who are usually responsive to fluoxetine antidepressant treatment [159]. Fluoxetine has shown anticancer potential in cell growth and tumour progression, inducing apoptosis and cell cycle arrest in different cancers [47], such as colorectal cancer [160], non-small-cell lung cancers [161,162] and hepatocellular carcinoma [162]. In AGS GC cells, fluoxetine inhibits cell proliferation. Additionally, apoptosis induced by fluoxetine is associated with an increase in activated caspases and PARP cleavage. The treatment with fluoxetine also increases the expression of the endoplasmic reticulum stress marker (CHOP), whose inhibition partially suppresses the apoptotic effect of fluoxetine by reducing the expression of death receptor 5, cleaved caspase 3 and cleaved PARP downregulation [47]. Furthermore, fluoxetine induces autophagosome formation and apoptosis simultaneously in AGS cells. The autophagy inhibitor 3-methyladenine improves fluoxetine-induced apoptosis by decreasing p-Akt and increasing the expression of death receptors 4 and 5 [48]. In sum, this drug reduces GC cell viability by inducing apoptosis through the upregulation of death receptors [47]. Fluoxetine was also evaluated in combination with paclitaxel in these cells, and was shown to promote an improvement in the antiproliferative effect of paclitaxel and an increase in apoptosis and to instigate necroptosis [49]. Therefore, fluoxetine may be a promising therapeutic agent for GC treatment [48].

Sertraline is an SSRI used in the treatment of major depression, anxiety and panic disorders, obsessive compulsive disorder, post-traumatic stress disorder, perimenopausal vasomotor symptoms, eating disorders, fibromyalgia and neuropathic pain [42,43,45,46]. In cancer, sertraline has shown antitumour activity in lung [163,164], prostate [165] and breast cancers [166], melanoma [167] and acute myeloid leukaemia [168]. A study proved that sertraline and its derivates can induce apoptosis and cell cycle arrest in the G0/G1 phase in SGC7901 GC cells resistant to cisplatin, through the phosphatidylinositol-3-kinase (PI3K)/Akt/ mammalian target of the rapamycin (mTOR) pathway [50]. The PI3K/Akt/mTOR signalling pathway plays an important role in cell growth and survival. The activation of this pathway leads to a perturbation in the control of cell proliferation and survival, resulting in a growth advantage, metastatic ability, angiogenesis and therapy resistance [169]. Therefore, sertraline and its derivates can be repurposed as chemosensitizers in drug-resistant GC treatment [50]. Paroxetine is an SSRI used in the treatment of the same disorders treated with fluoxetine and sertraline [42,43,45,46]. Besides its antidepressant activity, paroxetine exhibits anticancer features as it can induce apoptosis and suppress cancer cell proliferation in colon cancer [170], hepatocellular carcinoma [171], osteosarcoma [172] and lymphoma [173]. A study demonstrated that two GC cell lines present different sensitivities to paroxetine. AGS cells are more susceptible to this drug than MKN-45 cells. In AGS cells, paroxetine inhibits the expression of DNA repair proteins and increases DNA damage, inducing apoptosis and inhibiting cell proliferation. In addition, it promotes a decline in the mRNA and protein levels of Rad51, HR23B and ERCC1. Thus, improved DNA damage and reduced DNA repair enable paroxetine cytotoxicity in AGS cells. On the other hand, in MKN-45 cells, this drug cannot cause DNA damage or apoptosis [51]. The sensitivity differences between AGS and MKN-45 cells to paroxetine indicate that there is likely a genetic basis underlying this mechanism [51]. The combination of paroxetine and 5-FU, cisplatin, docetaxel or doxorubicin can induce a synergetic cytotoxic effect on MKN-45 cells [51]. This study indicates that paroxetine may be a potential anticancer agent or a sensitizer for chemotherapy and the synergistic outcome of the combination therapy can improve GC treatment [51].

#### 5.1.2. Antiepileptic Agents

Valproic acid (VPA) is a fatty acid that has antiseizure activity. VPA is an antiepileptic agent, and it is used in mania treatment [52,53,54]. VPA has proven anticancer characteristics in breast [174], prostate [175], cervical [176], oesophageal squamous [177], pancreatic and colon cancers [178]. VPA induces apoptosis and suppresses cell proliferation as well as the migratory capacity of the AGS GC cells [55]. In AGS and SGC-7901 GC cells, VPA suppresses histone deacetylase (HDAC) 1 and 2 activity and induces autophagy, which causes apoptosis through HDAC1/PTEN/Akt signalling pathway inhibition; it blocks the HDAC1/Atk signalling pathway but activates PTEN. This HDAC1/PTEN/Akt signalling pathway can play a critical role in cell proliferation, differentiation and immunogenicity. VPA also increases Beclin1 expression, demonstrating that GC cells experience autophagy-related cell death and activate intrinsic mitochondrial apoptosis, as well as blocking Bcl-2 expression [56]. Beclin1 functions as a tumour suppressor that has the ability to inhibit the malignant phenotype of GC cells, and was found downregulated in GC, being associated with poor prognosis [90]. Therefore, this Bcl-2-Beclin1 may be important in VPA-induced autophagy and cell death associated with autophagy. However, MKN-74 GC cells are resistant to VPA treatment, due to the high expression of HDAC1 protein and reduced expression of PTEN protein, suggesting that the evaluation of HDAC1 and PTEN expression can be used as a condition for GC treatment with VPA [56].

VPA’s effects were tested in vivo, where GC cell growth inhibition occurred through autophagy and apoptosis [56]. Regarding OCUM-2MD3, human scirrhous GC cells, VPA affects cell viability; increases the expression of acetyl-histone H3, acetyl-a-tubulin and p21WAF1; upregulates p27, caspase 3 and 9; and downregulates Bcl-2, cyclin D1 and survivin [57]. Survivin is a crucial protein in cell division and is able to inhibit cell death, being frequently upregulated in cancer [179]. In vivo, VPA reduced tumour volume and presented an increased apoptotic index [57]. In another study, BGC-823, HGC-27 and SGC-7901 GC cells were treated with VAP, experienced cell cycle arrest in the G1 phase due to the upregulation of P21Waf/cip1, Mad1 expression and downregulation of Cyclin A and c-Myc expression, and induced apoptosis mostly through the cytochrome C pathway (intrinsic pathway) by caspase 9 and caspase 3 activation [58]. An investigation demonstrated that AGS, BGC-823, NCI-N87 and MKN28 GC cells display higher levels of HDAC2 than normal cells, indicating that GC leads to an overexpression of HDAC2, which is associated with poor prognosis [59], as well as the overexpression of HDAC1 [56]. Moreover, VPA reduces HDAC2 levels, which suggests that VPA suppresses cell proliferation and induces apoptosis by inhibiting HDAC2 expression [59]. The combination of cisplatin with VPA has an improved effect when compared to cisplatin or doxorubicin alone, which indicates a synergistic effect. In vivo experiments also proved that VPA and cisplatin in combination with VPA decrease HDAC2 expression [59]. Nevertheless, a randomized phase II trial demonstrated that VPA does not offer a survival advantage when compared with paclitaxel alone in second- or third-line therapy for advanced GC [180].

#### 5.1.3. Statins

Statins consist of inhibitors of hydroxymethylglutaryl coenzyme A (HMG-CoA) reductase, which inhibits lipogenesis through the suppression of the mevalonate pathway, being used in cardiovascular disease treatment and being effective in reducing cholesterol [60,61,62,63].

Lovastatin has shown antitumour properties in different cancer cells, such as lung [181,182,183], ovarian [184], pancreatic [185], colon [186], thyroid [187,188], breast [189,190,191] and brain cells [192]. Lovastatin inhibits cell proliferation and induces apoptosis by repressing HDAC2 expression in AGS, BGC-823, NCI-N87 and MKN-28 GC cells. Furthermore, lovastatin in combination with cisplatin exhibits marked effects when compared to cisplatin or doxorubicin administration alone. Similar results have been shown in vivo, where HDAC2 suppression occurs due to lovastatin alone and cisplatin in combination with lovastatin. Therefore, lovastatin may be a potential drug for GC treatment [59]. In HGT-1 GC cells, lovastatin is able to repress the expression of genes associated with cell division and cell cycle progression, and also induce apoptosis. Lovastatin in combination with docetaxel improved the apoptotic effect. These data suggest that lovastatin alone or in combination with docetaxel may be a possible therapeutic approach for GC treatment [64]. A phase II study on high-dose lovastatin showed that this drug is well tolerated, but had no activity in patients with advanced GC [193].

Simvastatin, and lovastatin, is an inactive lactone prodrug which is hydrolyzed in the gastrointestinal tract to the active β-hydroxyl derivatives [62]. Simvastatin displays anticancer activity against different cancer types, such as colon [194,195], colorectal [196,197], breast [198], prostate [199] and lung cancers [200] and osteosarcoma [201]. In MKN-45 and MGC-803 GC cells, simvastatin promotes apoptosis and inhibits cell proliferation, migration and invasion. This drug also decreases β-catenin expression and its downstream targets c-Myc and cyclin D1, increases the LATS1 protein level and phosphorylation level of Yes-associated protein (YAP) at Ser127 and decreases the CYR61 levels, suggesting that simvastatin is able to inhibit YAP and β-catenin activity in GC cells [65]. The YAP oncoprotein is overexpressed in cancer due to YAP locus amplification or genetic/epigenetic inactivation of tumour suppressors [202]. In NCI-N87 (intestinal metastatic GC cells) and in Hs746T (diffuse metastatic GC cells), simvastatin affected cell proliferation differently, with Hs746T being more sensitive, indicating that simvastatin may be a potential drug for use in diffuse GC treatment [66].

Additionally, differences in response to simvastatin treatment have been found in AGS GC cells, with suppression of cell growth and decreased viability, but NCI-N87 GC cells offered more resistance to this drug [67]. In AGS, MKN-45, HGC-27 and MGC-803 GC cells, simvastatin displays an antitumour effect through the inhibition of cell proliferation and caspase-3/GSDME-mediated pyroptosis [68], a programmed gasdermin-mediated lytic cell death [203,204,205], with MGC-803 cells being the most sensitive and HGC-27 cells being the most resistant [68]. GSDME is a member of the GSDM family, which mediates pyroptosis through cleavage and activation by caspase-3. It also acts as a tumour suppressor by enhancing pyroptosis in response to chemotherapeutics that activate caspase-3 and turns out to be silenced in various tumours [203]. The re-establishment of GSDME expression by 5-Aza-CdR may be useful in sensitizing GC cells to simvastatin. In vivo results showed that the tumour growth suppression due to simvastatin can be improved by increased GSDME expression. Therefore, restoring GSDME expression and inducing pyroptosis might be a possible therapeutic approach in GC patients [68].

In SNU-5, SNU-16, MKN-45 and AGS GC cells, simvastatin inhibits cell proliferation. It induces downregulation of nuclear factor kappa B (NF-κB) and proteins controlled by NF-κB, such as cyclin D1, COX-2, survivin, Bcl-xL, XIAP, ICAM-1, MMP-9 and VEGF, playing an important role in GC cells’ proliferation, angiogenesis and metastasis [69], since the transcription factors of the NF-κB family regulate several genes, which are involved in cell proliferation, apoptosis, morphogenesis and differentiation [206], and therefore promotes cell damage response and cell survival [112]. Simvastatin in combination with capecitabine increases apoptosis and improves the previous effects [69]. In vivo experiments revealed that simvastatin alone and in combination with capecitabine inhibits tumour growth, being much more effective when used in combination. Therefore, simvastatin may be important in GC treatment, demonstrating improved effects when used with capecitabine [69]. Nonetheless, a placebo-controlled, double-blinded study demonstrated that simvastatin in combination with capecitabine–cisplatin in advanced GC does not increase progression-free survival [207]. A synergistic effect of simvastatin and radiation therapy on growth inhibition in GC cells (AGS, SNU601, MKN1 and MKN-28) was shown. Moreover, BIRC5 and CTGF may be important factors in this synergistic result, since simvastatin inhibits BIRC5 and CTGF expression, which improves the radiation effect on growth inhibition. In vivo experiments also corroborate the in vitro results, whereas the tumour growth is inhibited when simvastatin is combined with radiation therapy [70]. An investigation identified genes that are expressed in GC cells sensitive to the anticancer properties of simvastatin, such as TPK1 overexpression. TPK1 knockdown decreases the antitumour action of simvastatin, being a predictive marker of the station’s efficiency as an anticancer drug [208].

#### 5.1.4. Antipsychotic Agents

Antipsychotic agents, such as thioridazine and risperidone, are used in the psychiatric field to treat schizophrenia, bipolar disorder and acute mania [46,54,71,72,73].

Thioridazine exhibits antitumour features in glioblastoma [209], lung [210,211] and colon cancers [212]. In NCI-N87 and AGS GC cells, thioridazine has cytotoxic effects [74,75] through the inhibition of colony formation capacity and nuclear fragmentation and by inducing apoptosis in a caspase-dependent manner, decreasing the number of caspase-9, caspase-8 and caspase-3 precursors. In vivo, this drug is able to suppress tumour growth [74].

Risperidone inhibits the proliferation of KATO-III GC cells through the induction of apoptosis and increasing the reactive oxygen species level. This drug also suppresses tumour growth in vivo [76]. The anticancer effects of risperidone were demonstrated in a population-based cohort study, in which patients that used risperidone presented reduced risks of GC compared to non-users. However, further investigation is necessary [76].

#### 5.1.5. Angiotensin-Receptor-Blocking Agents

Angiotensin receptor blockers (ARBs), including telmisartan and candesartan, are used in hypertension, heart failure and diabetic nephropathy treatment [46,77,78].

The anticancer properties of telmisartan have been found in several cancers, such as prostate [213,214] urological (renal cell carcinoma, bladder and testicular cancers) [214], ovarian [215], colon [216], endometrial [217] and lung cancers [218], hepatocellular carcinoma [219], osteosarcoma [220], oesophageal squamous cell carcinoma [221] and melanoma [222]. In MKN-74, MKN1 and MKN-45 GC cells, telmisartan inhibits cell proliferation through cell cycle arrest in the G0/G1 phase by decreasing the expression of cyclin D1, cyclin-dependent kinase (CDK) 4 and the phosphorylation of the tumour suppressor retinoblastoma protein (Rb). In vivo data reveal that telmisartan suppresses tumour growth. Moreover, telmisartan can cause epidermal growth factor receptor (EGFR) phosphorylation inhibition and increase the levels of the angiogenesis-related protein tissue inhibitor of metalloproteinase-1 (TIMP-1) [79]. EGFR normal function involves the regulation of epithelial tissue development and homeostasis and can induce tumourigenesis in pathological conditions [223]. TIMP-1 is proven to be important in tumour aggressiveness and growth, and its overexpression is associated with poor prognosis in GC patients, as it reduces disease-free and overall survival [224]. Regarding the expression of miRNAs in MKN-74 cells treated with telmisartan, there was a decrease in miR-185-5p and miR-187 expression [79]. However, miR-185-5p is usually upregulated in GC and is a reliable biomarker in GC patients [225], and miR-187 is frequently overexpressed in GC, being associated with malignant features and promoting tumour growth and progression, and thus may be a prognostic marker and a potential therapeutic target in GC [226]. Telmisartan exhibits antitumour properties by arresting cell cycle and inhibiting the expression of miRNAs. However, the association between these two events demands further investigation [79].

Candesartan has been demonstrated to be effective in prostate [227], colorectal [228], lung [229] and bladder cancers [230] and in hepatocellular carcinoma [231].

In MKN-45 GC cells and an MKN-45 xenografted tumour model, candesartan decreased the expression of transforming growth factor β1 (TGF-β1) and suppressed the EMT and fibrosis in tumours [80]. TGF-β1 is associated with invasion, tumour progression, metastasis and EMT stimulation [232,233]. In samples from GC patients, increased expression levels of angiotensin II (Ang II) and angiotensin II type 1 and 2 receptors (AT1R and AT2R) have been found. An investigation demonstrated that in GC tumours in mice, Ang II enables tumour growth and in MKN-45 human GC cells, it increases proliferation and migration [234]. Results in vitro and in vivo show that candesartan inhibits Ang II-induced tumour growth [80]. Candesartan decreases TGF-β1 expression, EMT, tumour proliferation and fibrosis. Thus, combination therapy with angiotensin receptor blockers and antineoplastic agents can possibly improve prognosis and outcome in GC cases [80].

#### 5.1.6. Antidiabetic Agents

Metformin is the first-line therapy for diabetes type 2 treatment [46,60,61,81,82,83]. Additionally, it can be used in some syndromes with accompanied insulin resistance, including polycystic ovary syndrome, non-alcoholic fatty liver disease, gestational diabetes and some forms of premature puberty [46]. Metformin is also an agent with potential for repurposed chemotherapeutic use in some cancers, such as breast, colorectal, endometrial, ovarian, bladder, pancreatic and prostate cancers and primary brain tumours [82]. In GC, metformin is able to suppress cell proliferation, metastasis and stemness, promotes apoptosis and increases cells’ chemosensitivity [84]. In MKN1, MKN-45 and MKN-74 GC cells, proliferation is inhibited by metformin through cell cycle arrest in the G0-G1 phase, which is associated with a decline in cell cycle regulators (cyclin D1, CDK 4, CDK6 and a decrease in Rb phosphorylation). An in vivo experience also demonstrated that metformin suppresses tumour growth and decreases the expression levels of cell cycle regulators, indicating that metformin’s antitumour efficacy may be associated with a depletion of some cell-cycle-related proteins [85].

Another study confirmed that in GC cells (AGS, HR and TSGH), metformin represses cell growth and invasion skills. It also induces cell cycle arrest in the G2/M phase and decreases cell-cycle-related protein expression. Taken together, these data suggest that metformin inhibits GC growth by impairing cell cycle progression [86]. In AGS GC cells, metformin induces cell cycle arrest in the G0/G1 phase by decreasing CdK4 and CdK6 protein expression and also inhibits cell migration ability. The regulation of the cell cycle and cell proliferation depends on various proteins, such as 26S proteasome non-ATPase regulatory subunit 2 (PSMD2), adenylyl cyclase-associated protein 1 (CAP1) and stress-induced phosphoprotein 1 (STIP1). This drug also reduces PSMD2, CAP1 and STIP1 expression levels, which affect cell motility and migration. Moreover, patients that exhibit highly expressed PSMD2, STIP1 and CAP1 have a poor outcome [87]. In MKN-28, SGC-7901 and BGC-823 cells, metformin induces apoptosis by inhibiting survival [88]. The AMP-activated protein kinase (AMPK) and mTOR are associated with metformin activity [88]. AMPK is important in growth and metabolism regulation and autophagy [235]. AMPK knockdown enables the re-establishment of survival suppressed by metformin and consequently ceases metformin-induced apoptosis. Moreover, the overexpression of mTOR relieves survival inhibition and decreases apoptosis induced by metformin. Therefore, survival overexpression relieves metformin-induced apoptosis. In vivo results corroborate that AMPK/mTOR-mediated survival reduction is involved in apoptosis induced by metformin in GC cells [88].

The insulin-like growth factor-1 receptor (IGF-1R) plays a critical role in cellular growth and survival. In cancer development, it is involved in the metastatic process, regulating cell migration, invasion and angiogenesis [236]. EGFR and IGF-1R overexpression are usually associated with a poor outcome in GC [237]. In vitro and in vivo assays have shown that metformin decreases EGRF and IGF-1R phosphorylation and modifies miRNA expression. Thus, metformin inhibits GC cell proliferation and tumourigenesis through a decrease in cell cycle regulators induced by miRNA change. Since it is a widely used drug, metformin may be used as a novel agent for GC treatment via targeting cell cycle molecules [85]. MiR-107 acts as a tumour suppressor, inducing apoptosis and cell cycle arrest, and also promoting tumour growth and invasion. The expression of miR-107 is decreased in GC; however, its expression is upregulated in SGC-7901 GC cells treated with metformin. These data show that miR-107 enhances the antitumour properties of metformin in GC, preventing proliferation and invasion of those cells [89]. A study found that metformin decreased the expression of an oncogenic long noncoding RNA (lncRNA), Loc100506691, whose high expression levels are associated with a poor prognosis in GC patients. The knockdown of this lncRNA inhibits GC cell proliferation and induces cell cycle arrest in the G2/M phase. This lncRNA downregulates CHAC1 expression through the modulation of miR-26a-5p/miR-330-5p expression. Therefore, the antitumour properties of metformin in GC may be regulated by the Loc100506691-associated signalling pathway, which plays an important role in GC proliferation and migration by modulating the miR-26a-5p/miR-330-5p-CHAC1 axis pathway [86].

Besides the inhibition of the proliferation, migration and invasion of GC cells, metformin also promotes beclin1-dependent autophagy through the AMPK-mTOR signalling pathway [90]. Metformin plays an important role in tumour-associated fibroblasts, since it decreases the stimulatory effect of these cells on GC proliferation, impairing tumour growth through increasing calmodulin-like protein 3 secretion of tumour-associated fibroblasts. These data suggest a different anticancer mechanism of this drug [91]. Metformin is also able to induce cell cycle arrest and decrease cell proliferation and the number of tumourspheres in GC stem cells. In vivo results showed that this drug represses tumour growth and reduces the self-renewal capacity of cancer stem cells [92]. Another investigation found that lncRNA H19 is downregulated in GC cells previously treated with metformin. H19, which is associated with an advanced stage and the metastatic capacity of GC, is overexpressed in this disease. The H19 knockdown in AGS and SGC7901 GC cells reduces cell invasiveness, and, in this situation, metformin is not able to further decrease cell invasion after knockdown. Additionally, H19 reduction increases AMPK activation and decreases MMP9 expression, with metformin being unable to further activate AMPK or reduce MMP9. Thus, this drug displays anticancer properties in GC cells and H19 plays an important role in metformin invasion repression [93].

The EMT plays an important role in metastasis, and its markers can be inhibited by some drugs or siRNA. Vimentin is upregulated in the EMT. AGS GC cells treated with metformin and vimentin-specific siRNA (vim-siRNA) showed that vimentin inhibition due to metformin was similar to vim-siRNA, with a decrease in cell migration and invasion ability. Thus, these results suggest that metformin can be used as a substitute for specific siRNAs for vimentin expression inhibition and migration of GC cells [94]. Metformin is able to inhibit the Shh signalling pathway in GC cells (HGC-27 and MKN-45) in an AMPK-dependent manner. This drug decreases the mRNA levels of Shh and Glioma-associated oncogene (Gli)-1 and inhibits the gene and protein expression levels of Smoothened (SMO), Gli-2 and Gli-3, and the reduction in AMPK induced by the small interfering RNA reverses the inhibitory effect of metformin on Shh-induced expression of Gli-1 in GC cells [95]. An investigation found that coiled-coil domain containing 65 (CCDC65) downregulation is a negative factor in GC patients. In vitro and in vivo data show that CCDC65 is a GC growth and metastasis inhibitor. The binding of CCDC65 to the oncogenic factor ENO1 stimulates tumour pathogenesis and promotes ubiquitylation and degradation of ENO1 through the recruiting of E3 ubiquitin ligase FBXW7. The downregulation of ENO1 reduces the binding with AKT1, which is inactivated, and inhibits cell proliferation and loss of EMT signals. Metformin leads CCDC65 to repress ENO1-AKT1-complex-mediated cell growth and EMT signals, and thus inhibits the malignant GC phenotype, indicating another molecular mechanism of GC suppression by metformin [96].

Metformin can induce apoptosis and suppress invasion and migration of GC cells through the inhibition of HIF1α, whose overexpression is associated with increased GC cell viability and invasion. High expression levels of pyruvate kinase M2 (PKM2) were found in GC samples, which suggests that glycolysis is involved in GC development. However, metformin is also able to inhibit PKM2 expression. Therefore, this drug displays anticancer features in GC by inhibiting the HIF1α/PKM2 pathway [97]. A study demonstrated that GC cells’ (MKN1, KATO-III and SNU-1) sensitivity to metformin increases at low glucose levels in vitro. In vivo experiments showed that this drug is able to suppress peritoneal metastasis of GC through the inhibition of the NF-ĸB signalling pathway. However, no changes have been reported in the AMPK pathway, which is the critical target signalling pathway of metformin. Thus, metformin’s suppression of peritoneal metastasis of the GC and NF-ĸB pathway plays an important role in this mechanism [98].

In AGS GC cells, metformin suppresses EMT-related genes, decreasing mesenchymal markers (vimentin and β-catenin) and promoting epithelial markers (E-cadherin), and also inhibits migration and invasiveness under hyperglycaemic conditions [99]. The combination of metformin and cisplatin in MKN-45 GC cells showed that metformin decreases the antiproliferative action of cisplatin. When both drugs were used in combination, the survivin and mTOR expression increased, indicating that the antagonistic effect of metformin and cisplatin is induced through survivin and mTOR signalling pathways. Therefore, metformin is not a good choice to sensitize GC cells to cisplatin, and the co-administration of both drugs in GC patients with diabetes type 2 should be assessed [238].

#### 5.1.7. Antibiotics

Doxycycline belongs to the tetracycline antibiotics family and exhibits activity against a wide range of Gram-positive, Gram-negative and “atypical” bacteria. It is used worldwide in the treatment of sexually transmitted diseases, respiratory tract infections, malaria prophylaxis and rickettsial infections [46,100,101,102]. Doxycycline acts through bacterial protein synthesis inhibition by reversibly binding to the 30S ribosomal subunit and preventing the connection of aminoacyl-tRNA with the bacterial ribosome [100,239]. Moreover, doxycycline can prevent angiogenesis and apoptosis, and inhibit matrix metalloproteinase (MMP) activity [46,100,239], which is produced by inflammatory cells. Thus, doxycycline displays anticancer and anti-inflammatory properties [100,239]. Some investigations reported its anticancer features in prostate [240], cervical [241] and duodenum cancers [242].

The mitogen-activated protein kinase (MAPK) pathways connect the extracellular signals to the controlling mechanisms of the cellular processes, such as growth, proliferation, differentiation, migration and apoptosis. ERK is a member of the MAPK pathways and is frequently deregulated in human cancers, which is related to cell proliferation [243]. Doxycycline is able to inhibit ERK/MAPK activity on transcriptional and protein levels. In AGS, MKN-45 and KATO-III GC cells, doxycycline effectively inhibits cell proliferation, colony formation and spheroid growth characteristics. The propensity for ERK/MAPK inhibition due to doxycycline is greater in KATO-III cells, followed by MKN-45 and AGS cells, which may be a result of the activation of other transcription factors or signalling pathways, being important combinatory targets of pathways along with the inhibition of ERK/MAPK [244]. The genome-wide mRNA and transcription factor profiling showed that doxycycline inhibits oncogenic ER, Myc, E2F1, Wnt and SMAD2/3/4 pathways and activates NRF1/2, MTF1 and ATF6, stress-related pathways, and the p53-mediated tumour suppressor pathway. Doxycycline also inhibits HSP90 and RPS6KA1, upstream and downstream factors involved in the ERK/MAPK pathway, suggesting that it may be an option for targeting the proteasomal pathway and molecular chaperones [244]. In AGS cells, doxycycline downregulates the proteasomal pathway, ubiquitin ligation, DNA polymerases and histone genes. Since doxycycline is able to inhibit various oncogenic pathways and is already used as a broad-spectrum antibiotic, it may be a potentially useful and safe chemotherapeutic agent in GC therapy [244].

Tigecycline is a glycylcycline approved for skin and soft-tissue infection, intra-abdominal infections, and community-acquired pneumonia treatment. However, this drug is associated with an increased risk of death when compared to other antibiotics used to treat these infections [101,103]. Tigecycline exerts its antitumour effects on different cancer types, such as lung [245,246], thyroid [247], pancreatic [248], glioma [249], melanoma [250], chronic myeloid leukaemia [251], multiple myeloma [252], neuroblastoma [253], oral squamous cell carcinoma [254], cervical squamous cell carcinoma [255] and lymphoma [256]. In GC, Tigecycline inhibits cell (GAM-016 and MKN-45) proliferation and is able to induce autophagy through the activation of the AMPK pathway and suppression of its downstream targets, such as mTOR and p70S6K. Thus, tigecycline may be a potential drug for GC therapy [104].

#### 5.1.8. Antialcohol Agents

Disulfiram is an inhibitor of aldehyde dehydrogenase used in alcohol abuse treatment [46,105,106]. Disulfiram exhibits antitumour properties, such as inhibition of cancer cell stemness, proteasome activity and metastasis; increased reactive oxygen species; formation of a complex with metal ions; and DNA methylation suppression [257]. This drug has been proven to be effective in various cancer types, including non-small-cell lung cancer [258], liver [259], breast [260,261,262], ovarian [263] and pancreatic cancers [264] and glioblastoma [265]. Disulfiram suppresses MKN-45 and SGC-7901 GC cell proliferation, and the results showed that it inhibits the proliferating cell nuclear antigen (PCNA) and MMP-2 expression [107]. PCNA is a cell cycle marker, being important in nucleic acid metabolism for functions such as DNA replication, DNA excision repair, cell cycle control, chromatin assembly and RNA transcription [266]; MMP-2 is a biomarker for migration and invasion [107]. Disulfiram also downregulates the Wnt and NF-κB signalling pathways [107]. The Wnt signalling pathway is related to cellular processes during development, such as cell differentiation, polarization and migration. Thus, its deregulation is involved in cancer biology [267].

A study demonstrated that disulfiram chelated with copper inhibits MKN-45 and BGC-803 GC cell migration and invasion, suggesting that it is able to repress tumour growth and metastasis. In vivo experiments revealed that this treatment induces autophagy and increases autophagy-related Beclin1 and light-chain (LC) 3 protein expression. It also promotes oxidative stress and DNA damage by increasing reactive oxygen species, elevating P53, P21 and g-H2AX proteins and repressing the Wnt/β-catenin signalling pathway [268]. Another investigation assessed the efficiency of disulfiram chelated with copper in HGC-27 and SGC-7901 GC cells, which showed an inhibition in proliferation and growth. It also raises reactive oxygen species and promotes apoptosis in a reactive oxygen species-dependent manner [269]. Therefore, disulfiram may be a promising therapeutic approach for GC treatment [107,268].

#### 5.1.9. Iron Chelator Agents

Deferasirox is a tridentate iron chelator approved for treatment of iron overload caused by blood transfusions, thalassemia and myelodysplastic syndrome [108,109,110]. Several studies reported that deferasirox displays antitumour activity in various malignancies, such as pancreatic [270,271,272,273] and breast cancers [274], mantle cell lymphoma [275] and lymphoma [276]. Deferasirox inhibits AGS, MKN-28, SNU-484 and SNU-638 GC cell growth. AGS cells treated with deferasirox experience cell cycle arrest in the G1 phase, through the upregulation of p21, p27 and p53 expression, and downregulation of cyclin D1, cyclin B and CDK4 expression. It was also found that deferasirox induces apoptosis and upregulates N-myc downstream regulated gene 1 (NDRG1) [111], which plays an important role in cancer progression by inhibiting metastasis [277], and downregulates phospho-mTOR and c-myc expression, showing that this drug exhibits antitumour effects on GC cells through different pathways [111]. The combination of deferasirox and cisplatin also led to a reduction in cellular viability in the upregulation of NDRG1, p21 and p53 and the downregulation of phospho-mTOR. Such results indicate that deferasirox can possibly improve the antitumour effect of cisplatin in GC and that the NDRG1, p21, p53 and mTOR pathways may be associated with the synergistic effect of this drug combination [111].

#### 5.1.10. Proteasome Inhibitors

Bortezomib, a boronic acid dipeptide, is a first-generation proteasome-mediated protein degradation inhibitor, approved as a first-line therapy in multiple myeloma (MM) and mantle cell lymphoma (MCL) and also in relapsed or refractory MM and MCL after prior therapy with other drugs [26,46,112,113]. Bortezomib inhibits the 26S proteosome, leading to a downregulation of the NF-κB signalling pathway [26]. The majority of cellular NF-κB is cytosolic and bound to IκB, being restricted to the cytosol and unable to regulate transcription. However, IκB is ubiquitinated and then degraded through the proteasome in response to hypoxia, chemotherapy and DNA damage, which releases NF-κB that enters the nucleus and transcriptionally activates genes associated with cell survival (cell adhesion proteins), proliferation (cyclin D1) or antiapoptosis (cIAPs, BCL-2). NF-κB has been found to be highly expressed in some tumours, such as MM, playing an important role in tumour cell survival in a hypoxic environment and throughout chemotherapy treatment [112]. Proteasomal degradation of IκB is blocked by bortezomib, which prevents the NF-κB transcriptional activity, downregulates survival responses and disrupts the ubiquitin–proteasomal degradation of p21, p27, p53 and apoptosis initiators [112].

Bortezomib can inhibit tumour cell proliferation and induce apoptosis in SNU638, MUGC-3 and MKN-28 GC cells. In SNU638 cells, bortezomib produces a decline in BCL-2 protein expression, being even further decreased by the combination therapy of bortezomib plus docetaxel, implying that together they can downregulate BCL-2 expression. However, in these cells, bortezomib increased BAX (pro-apoptotic protein) levels, and when used in combination with docetaxel, there was a notorious increase in this protein, displaying a synergetic effect [114]. Neither bortezomib alone nor in combination with docetaxel exhibits a positive result on p53 protein expression levels, meaning that bortezomib inhibits cell proliferation and promotes apoptosis in a p53-independent manner in SNU638 p53 mutant GC cells. This fact reinforces the importance of this drug, which acts independently of the p53 mutational tumour’s status. Bortezomib stabilizes p27, demonstrating that this protein may be associated with the antitumour activity of this drug, and when used in combination with docetaxel, there was an increase in the p27 stabilization level [114]. Bortezomib suppresses GC cell growth by inducing apoptosis, and the combination treatment of bortezomib and docetaxel leads to an improvement in cytotoxicity and apoptosis in GC cells, suggesting it may be a potentially effective drug for GC treatment [114].

#### 5.1.11. Nonselective β-Adrenergic Receptor Antagonist

Propranolol is a nonselective β-adrenergic receptor antagonist (β-blocker) that blocks β1 and β2 adrenergic receptors equally [78,82,115,152,278]. It is effective in hypertension, angina pectoris, arrhythmias, migraine, hyperthyroidism, anxiety and tremor treatment [78,115]. In oncology, propranolol exhibits effects on cellular proliferation and invasion, the immune system, the angiogenic cascade and cells’ sensitivity to existing treatments [82]. It has been proven to be useful in infantile haemangiomas and angiosarcoma treatment [82,152]. Propranolol inhibits GC cell proliferation [116,117]. It induces apoptosis through β-adrenergic receptors pathways in BGC-823 and SGC-7901 GC cells [116] and also inhibits cell growth in BGC-823 GC, MKN-45 and NUG-3 cells due to cell cycle arrest in the G1 phase [116,117] in SGS-7901 GC cells through cell cycle arrest in the G2/M phase [116]. Additionally, propranolol reduces cyclin D1, E2 and phosphorylated Rb expression, which is implicated in the cell cycle G1/S phase transition [117].

The cell proliferation and invasion induced by β-adrenergic receptors is in part due to NF-κB pathway activation, since this upregulates some target genes, such as cyclooxygenase-2 (COX-2), MMPs and vascular endothelial growth factor (VEGF). Propranolol, through β-adrenergic receptors, decreases NF-κB DNA-binding activity, leading to inhibition of Cox-2, MMP-2/9 and VEGF expression [116]. These findings suggest that propranolol displays antiproliferative and antimetastatic properties, since it represses cell invasion, migration and angiogenesis through GC cell growth suppression by inhibiting β-adrenergic receptors, decreasing NF-κB levels and downregulating MMP-2/9, Cox-2 and VEGF gene expression [116,117]. In vivo, this drug showed antitumour activity in an MKN-45 xenograft mouse model, since it suppresses cell proliferation and induces apoptosis, and is thus a potential candidate for GC treatment [117].

#### 5.1.12. α1-Adrenoceptor Blockers

α1-adrenoceptor antagonists, such as naftopidil, are usually used for their vasodilator effects in cardiovascular diseases and in prostatic hyperplasia treatment [46,118]. Naftopidil has been shown to be effective in prostate [279,280,281,282] and ovarian cancers [283], renal cell carcinoma [284] and malignant pleural mesothelioma [285]. Naftopidil decreases cell viability and induces apoptosis in HGC27 GC cells. This drug decreases AKT phosphorylation, suggesting that apoptosis is induced through PI3K/AKT pathway inhibition. Naftopidil also promotes autophagy due to the increase in LC3-II and the shift from LC3B to autophagosomes. Besides the antitumour effect it exhibits due to apoptosis induction, naftopidil in combination with chloroquine diphosphate, an autophagy inhibitor, displays a synergistic anticancer effect [119].

The synthesized naftopidil analogue, HUHS1015 (1-[2-(2-Methoxyphenylamino)ethylamino]-3-(naphthalene-1-yloxy)propan-2-ol), reduces cell viability in MKN-28 and MKN-45 GC cells, being even more effective than cisplatin, and also induces necrosis and apoptosis. However, apoptosis is promoted through different mechanisms in MKN-28 and MKN-45, since HUHS1015 activates caspase-3, caspase-4 and caspase-8 in MKN-45, but no such activation was found in MKN-28 cells [120]. The in vivo results showed that HUHS1015 represses tumour growth; however, naftopidil has no significant effect, suggesting that HUHS1015 is more effective in GC cells than naftopidil. The survival rate for HUHS1015, cisplatin, paclitaxel and irinotecan was 100%, 43%, 86% and 71%, respectively [120]. This drug has also been shown to be effective in malignant pleural mesothelioma [286]. This naftopidil analogue, HUHS1015, induces apoptosis in a caspase-independent and caspase-dependent manner in MKN-28 and MKN-45 GC cells. It also inhibits tumour growth in vivo with an improvement in survival rate when compared to other anticancer drugs, and is thus a potential candidate for GC treatment [120].

#### 5.1.13. Aurora Kinase Inhibitors

Aurora kinases consist of a group of serine/threonine kinases that regulate mitotic cellular division, being important for cell cycle control [287,288,289]. Aurora kinase A (AURKA) is associated with centrosome function, mitotic entry and spindle assembly, and aurora kinase B is involved in chromatin modification, microtubule–kinetochore attachment, spindle checkpoint and cytokinesis [288]. Furthermore, this kinase family plays an important role in tumourigenesis, since its overexpression has been reported in some tumours [287]. AURKA is a viable target in cancer therapy, since its overexpression is common in tumours [290,291], and it has been reported in upper gastrointestinal adenocarcinomas, with the AURKA/AKT axis being an important pathway for enhancing the antiapoptotic characteristics of cancer cells through the regulation of p53-dependent apoptosis [290]. In GC patients with curative surgery, AURKA has been proven to be a molecular prognostic marker, and is therefore a valuable factor in individualized treatment [292].

Danusertib is a small-molecule 3-aminopyrazole derivative which inhibits the adenosine triphosphate (ATP) site of all members of the aurora kinase family (aurora A, B and C) [293,294]. It also displays cross-reactivity with other kinases, including Ret, TrkA, FGFR1 and Abl, which may be important in danusertib anticancer activity, since the oncogenic Bcr-Abl tyrosine kinase causes chronic myelogenous leukaemia and BCR-ABL-positive acute lymphocytic leukaemia development [294], being a third-generation Bcr-Abl tyrosine kinase inhibitor with potent anticancer effects [121]. Danusertib exhibits antiproliferative action in various cancer cells, accumulating tetraploid cells in G1-like growth arrest. This drug decreases histone H3 phosphorylation and aurora A autophosphorylation, being an effective inhibitor of both kinases [293]. Various studies have shown antitumour activity of danusertib in melanoma [295], prostate [296], breast [297] and ovarian cancers [298], hepatocellular carcinoma [299] and leukaemia [300].

In AGS and NCI-N78 GC cells, danusertib arrests cell cycle in the G2/M phase with a reduction in cyclin B1 and CDC2 expression, an important complex in G2/M phase transition and mitosis. This drug also increases p53, p27 Kip1 and p21 Waf1/Cip1 expression, supporting cell cycle arrest in the G2 phase [121]. Danusertib induces mitochondria-dependent apoptosis, since it promotes cytochrome c release from mitochondria to the cytosol, which activates caspase 9 and caspase 3, leading to apoptosis induction by Bcl-2 decreased level. Moreover, it induces autophagy through PI3K/Akt/mTOR signalling pathway inhibition, and AMPK and p38 MAPK play important roles in danusertib-induced autophagy [121]. The EMT is inhibited due to this drug’s action in increasing E-cadherin expression and reducing N-cadherin expression [121]. Danusertib exhibits its effects by arresting the cell cycle, inducing apoptosis and autophagy and inhibiting the EMT, involving different signalling pathways in GC cells [121].

Alisertib is a selective small-molecule inhibitor of AURKA that has been tested for various haematologic and non-haematologic malignancies [289,291]. This agent may have beneficial effects over traditional chemotherapy, such as simpler dosing, less toxicity and potentially improved outcomes in different cancers. Alisertib in combination with cytotoxic chemotherapy has also been investigated to evaluate which cancer types might benefit from this therapy [289]. Alisertib is able to induce cell cycle arrest, apoptosis, autophagy, polyploidy and mitotic catastrophe and also induce tumour regression alone or in combination with other anticancer drugs [291]. In colorectal [301], breast [302,303] and small-cell lung cancers [303], hepatocellular carcinoma [304] and leukaemia [305], alisertib has shown antitumour effects. Alisertib improves the anticancer properties of some cytotoxic drugs, including taxanes, cisplatin and vincristine [291].

In AGS and NCI-N78 GC cells, alisertib inhibits cell proliferation and induces cell cycle arrest in the G2/M phase, which is regulated by CDC2 and cyclin B1. Alisertib decreases expression of these two regulators in AGS and NCI-N78 cells, and increases p53, p27 Kip1 and p21 Waf1/Cip1 expression, which might aid the cell proliferation suppression and cell cycle arrest by this AURKA inhibitor. It also enables mitochondria-dependent apoptosis, since it increases the cytosolic level of cytochrome c, which in turn activates caspase 9, which activates caspase 3 and induces apoptosis with a reduction in Bcl-2 expression, a proapoptotic protein. Additionally, alisertib induces autophagy through PI3K/Akt/mTOR signalling pathway inhibition, which potentiates the antitumour properties. This AURKA inhibitor activates AMPK, a cell death regulator, and represses p38 MAPK phosphorylation, suggesting that AMPK and p38 MAPK are important in autophagy induced by alisertib in GC cells [122]. Alisertib suppresses cell proliferation, promotes cell cycle arrest and autophagy, and activates the mitochondria-dependent apoptotic pathway in GC cells through PI3K/Akt/mTOR, p38 MAPK and AMPK signalling pathways [122].

AURKA and HDM2 have been found to be overexpressed in GC cells in about half of the primary GC tumours [123]. HDM2 consists of an E3 ubiquitin ligase that inhibits p53 activity [306]. p53 is a transcriptional factor that induces pro-apoptotic proteins and suppresses cell growth, playing an important role in cancer [123,307,308]. Therefore, both HDM2 and AURKA are essential in p53 regulation [123]. A study using AGS and SNU-1 GC cells reported that AURKA overexpression increases HDM2 protein levels and downregulates p53 and p21. On the other hand, alisertib decreases HDM2 levels and upregulates the expression of p53 and p21, suggesting that in GC cells, AURKA positively regulates HDM2. Furthermore, AURKA overexpression reduces the induced expression of p53 and p21 by cisplatin, which indicates that AURKA possibly intervenes in resistance to cisplatin by HDM2/p53 signalling regulation [123]. In vivo experiments have shown that AURKA inhibition by alisertib decreases tumour growth and HDM2 expression, and induces p53 and its downstream transcriptional targets. Thus, AURKA-HDM2 may be a potential therapeutic target of AURKA inhibitors in GC treatment [123].

#### 5.1.14. Nucleoside Reverse Transcriptase Inhibitors

Abacavir is a nucleoside reverse transcriptase inhibitor (NRTI), a guanosine analogue approved for the treatment of human immunodeficiency virus (HIV). It is associated with hypersensitivity skin reactions, due to the HLA variant HLA-B*5701 [46,124,125,126]. Abacavir is a prodrug that demands intracellular phosphorylation to generate carbovir triphosphate, its active metabolite. Carbovir triphosphate can inhibit viral reverse transcriptase through competition with endogenous deoxyguanosine triphosphate. This metabolite induces DNA chain termination since it lacks the 3′ hydroxyl group, which is required to form the phosphodiester bonds, suppressing the reverse transcription process [309]. Abacavir displays anticancer activity in prostate [310] and oesophageal squamous cancers [311] and medulloblastoma [312]. YY1 is a transcriptional factor that plays an important role in cell growth, apoptosis, tumourigenesis, embryonic development and differentiation [313,314]. YY1 is involved with both stimulation and suppression of tumour growth, being overexpressed in several cancer types and associated with a poor outcome [314,315]. Therefore, it is a prognostic marker for various tumours and a therapeutic target for cancer therapy [313]. In GC, YY1 regulated genes were found to be highly expressed, being also associated with a poor prognosis and outcome of those patients. A study showed that abacavir does not significantly reduce AGS GC cells’ viability, since 40% of these cells were still viable after 48 h. However, abacavir activates oncogenic signalling pathways in AGS cells, such as β-catenin, MYC, WNT, ERK/MAPK, NRF1/2, NFκB, HIF1α and YY1, which may be a sign of cellular senescence [127].

The experiments also demonstrated a co-occurrence of the activated TERT and DNA repair processes, and a co-activation of ALT and various oncogenic pathways, including E2F/DP1, MYC, YY1, ERK, NFκB, HIF1α and WNT, revealing that TERT inhibition alone is not enough to suppress cancer cells’ growth. These pathways may also be involved in drug resistance. Furthermore, abacavir activates the YY1 transcription factor and other oncogenic pathways without TERT interference [127]. Thus, abacavir activates different pathways in GC cells and co-activates TERT, ALT and DNA repair, indicating that combination therapy could be more effective than single-agent drugs [127].

#### 5.1.15. Anaesthetic Agents

Propofol is an alkyl phenol with hypnotic characteristics frequently administered for induction and maintenance of general anaesthesia and maintenance of long-term sedation [128,129,130]. Besides its sedative properties, propofol also exhibits anticancer activity [316] in gallbladder [317], breast [318,319], colon [320] and lung cancers [321], hepatocellular carcinomas [322] and osteosarcoma [323]. It also induces apoptosis in lung cancer [321] and osteosarcoma [323]. In GC patients anesthetized with propofol, an improvement in the cytotoxicity of natural killer (NK) cells has been reported, enhancing the tumour-cell-killing function of these cells [324]. In SGC7901 and AGS GC cells, propofol inhibits cell proliferation and invasiveness, promotes apoptosis and also decreases miR-221 expression. An increase in Mi-R221 is associated with cell proliferation and invasion and apoptosis inhibition, acting as an oncogene. Therefore, a decrease in miR-221 expression may contribute to the anticancer properties of this drug [131]. Other studies demonstrated that in SGC-7901 and MGC-803 GC cells, propofol suppresses cell proliferation, invasion and migration and promotes apoptosis [132,133]. In these cells, inhibitor of growth 3 (ING3) expression is lower than that in normal GC cells, suggesting that its downregulation is associated with GC carcinogenesis. Propofol increases ING3 expression; thus, ING3 is associated with the inhibitory effect of this drug. Therefore, propofol can suppress GC progression and ING3 may be a potential therapeutic target [132].

In MKN-45 GC cells, propofol also inhibits proliferation, migration, invasion and promotes apoptosis. This drug upregulates miR-195 expression and inactivates the Janus kinase (JAK)/ signal transducer and activator of transcription (STAT) and NF-κB pathways, while miR-195 suppression reverses propofol-induced inhibition of cell proliferation, migration and invasion, induction of apoptosis and JAK/STAT and NF-κB pathway inactivation. Furthermore, JAK/STAT and NF-κB pathway inhibition reverses the inhibitory effects of miR-195 on propofol-induced cell proliferation, migration and invasion inhibition and apoptosis. Thus, propofol displays antineoplastic properties in GC cells by inhibiting cell proliferation, migration and invasion, inducing apoptosis and inactivating JAK/STAT and NF-κB pathways through miR-195 upregulation [134]. In GC, propofol improves cisplatin sensitivity by inhibiting the chemoresistance of GC cells by reducing autophagy-related chemoresistance to cisplatin through the inhibition of lncRNA metastasis-associated lung adenocarcinoma transcript 1 (MALAT1). Therefore, propofol in combination with cisplatin may be effective in chemoresistance inhibition of GC cells, targeting autophagy and improving prognosis [325]. Ferroptosis regulation is another function of propofol in GC. This drug induces ferroptosis and inhibits malignant phenotypes of GC through the regulation of the miR-125b-5p/ signal transducer and activator of the transcription 3 (STAT3) axis, proving its potential for GC therapy [133].

Levobupivacaine is a local anaesthetic drug that causes a reversible blockade of open neuronal sodium channels, producing analgesia and anaesthesia. The local anaesthetic agents are used in pain control during labour, in postoperative periods and as a relief therapy in patients with chronic pain [135]. Several studies have reported the antitumour effects of levobupivacaine in different cancer types, such as breast [326,327,328], prostate [329] and colon cancers [330], melanoma [327] and osteosarcoma [331]. Levobupivacaine inhibits cell proliferation of GC cells in vitro and in vivo. Additionally, it induces ferroptosis by upregulating miR-489-3p and targeting SLC7A11. Besides ferroptosis, miR-489-3p is known to suppress cell proliferation, migration, invasion and glycolysis in cancer. Therefore, levobupivacaine may be a potential antitumour agent in GC [136].

#### 5.1.16. Immunosuppressive Agents

Mycophenolic acid is the active form of the mycophenolate mofetil drug. Mycophenolic acid inhibits inosine monophosphate dehydrogenase, suppressing T- and B-lymphocyte proliferation [46,152,332]. Moreover, this drug may improve apoptosis [152]. Mycophenolic acid has been shown to be effective in osteosarcoma [333]. In AGS GC cells, mycophenolic acid induces cell cycle arrest in the G1/S phase and cell proliferation inhibition due to the inhibition of the PI3K/AKT/mTOR pathway and causes caspase-dependent apoptosis through the upregulation of the p53 and FAS pathways. These data indicate that mycophenolic acid displays antitumour action via targeting different cell processes [137].

Rapamycin is a product from a bacterium, *Streptomyces hygroscopicus.* It inhibits the mTOR, a serine/threonine protein kinase, which is involved in cell cycle progression, cell proliferation, angiogenesis and metabolism [82,112,334]. The signalling pathways upstream and downstream of the mTOR are often dysregulated in cancer, and its functions depend on the mTOR-associated complexes’ activity, such as mTORC1 and mTORC2 [82]. Rapamycin inhibits mTORC1, an enzyme complex that is in a downstream position in the PI3K pathway [112]. The mTORC1 complex is formed with FKBP12, a member of the FK506-binding protein family [82,112]. The anticancer properties of this drug come from its binding to FKBP12 and inhibition of mTOR activation [82,112,334]. Rapamycin and its relatives are the first-line drugs used in post-transplant immunosuppression. It has also been approved for renal cancer treatment; neuroendocrine tumours of the pancreas, gastrointestinal tract and lung; and for antihormone refractory and hormone-receptor-positive breast cancer [112]. Rapamycin inhibits cell proliferation in TMK-1, MKN-28, MKN-45 and MKN-74 GC cells and induces cell cycle arrest in the G1 phase. The combination therapy of rapamycin, docetaxel, cisplatin and 5-FU has shown an increase in sensitivity, indicating that rapamycin may improve the cytotoxicity of each chemotherapeutic drug. In TMK-1 cells, rapamycin in combination with docetaxel or cisplatin increased apoptosis levels compared with those drugs alone, suggesting that rapamycin improves docetaxel’s and cisplatin’s effect through increased apoptosis levels [138]. Regarding the cell growth signal transduction pathways, rapamycin in combination with docetaxel reduced the phosphorylation of ERK1/2 and mTOR-4E-BP1 pathways and increased activation of the apoptotic pathway in TMK-1 cells [138]. These data suggest that rapamycin, an mTOR inhibitor, can improve the effects of other drugs used in GC treatment [138].

#### 5.1.17. Photosensitizing Agents

Verteporfin is a benzoporphyrin derivative, a photosensitive (light-activated) drug approved for photodynamic therapy of choroidal neovascularization associated with macular degeneration [139,140]. Regarding its antitumour properties, it has shown positive results in pancreatic [335], endometrial [336], ovarian [337,338,339], bladder [340], cervical [341], breast [342], colon [343] and papillary thyroid cancers [344], glioblastoma [345], mesothelioma [346] and melanoma [347]. The Hippo pathway is involved in cell proliferation, organ size control and cancer development/progression [348]. The transcriptional coactivator with the PDZ-binding motif (TAZ) binds to transcription factors in order to control cell differentiation and organ development [348,349] and is inhibited by the Hippo pathway [348]. YAP/TAZ consists of downstream effectors of the Hippo pathway, and it plays an important role in cancer, since tumours may benefit from its properties, which aid in proliferating, migrating and metastasizing. YAP/TAZ’s high gene expression and aberrant nuclear location are associated with poor outcomes [350]. The TEAD family transcription factors also mediate YAP/TAZ biological activity [348]. In GC, YAP1 overexpression is associated with progression, metastasis and poor prognosis, being a possible diagnostic and prognostic marker for GC patients [351,352]. YAP also decreases sensitivity to cisplatin of GC cells by targeting EGFR signalling, which suggests the YAP/EGFR axis as a potential target for GC therapy [353]. TAZ was found to be highly expressed in gastric signet ring cell carcinoma, and thus is a potential target for this cancer treatment [349]. The high expression levels of YAP and TAZ in GC cells was found to be associated with a poor prognosis and outcome [141]. Thus, YAP inhibitors may be potential drugs for cancer treatment. Verteporfin is a transcriptional inhibitor of YAP1 and TEAD interaction, repressing the YAP1-TEAD complex oncogenic action [202]. Verteporfin inhibits AGS, NCI-N87, MKN1, SNU638 [142], MKN-45 [141,142,143] and MKN-74 GC cell proliferation [141,143].

In vivo assays demonstrated that verteporfin reduces tumour growth. These data indicate that verteporfin could be used in GC treatment [142]. FAT atypical cadherin 1 (FAT1) encodes a protocadherin, a gene frequently mutated in cancer, and it can act as an oncogene or a tumour suppressor gene, depending on the cancer type [354]. In GC samples, FAT1 expression was found to be highly upregulated, being associated with a worse prognosis in those patients. However, the loss of FAT1 leads to a decrease in GC cell growth, which indicates that targeting FAT1, when it displays oncogenic activity, may be a potentially useful treatment strategy in GC [142]. Another investigation found that clusterin is important for GC stem cells’ survival due to regulating HSP90 action [144]. Clusterin is a molecular chaperone that exists in various tissues and controls cancer cell proliferation through the inhibition of programmed cell death, stemness, metastasis, the EMT and therapy resistance [355]. Verteporfin inhibits the clusterin gene expression in those GC stem cells and suppresses its viability and growth in vivo, indicating that clusterin-targeted therapy and verteporfin therapy may be useful in repressing cancer growth by HSP90 function regulation [144]. A study showed that YAP1/TAZ-TEAD and its target genes are composed of a cluster of differentiation-44 (CD44)-positive cells and upregulated in resistant cells after chemotherapy treatment. Verteporfin is able to inhibit YAP1/TAZ-TEAD activity, cancer stem cell markers’ expression and the tumoursphere in GC, which allows tumour growth inhibition both in vitro and in vivo. Thus, verteporfin may be a potential cancer-stem-cell-based approach in GC therapy targeting YAP1/TAZ-TEAD activity [356].

Apoptosis is also induced by verteporfin treatment through the production of singlet oxygen molecules, reactive oxygen species and mitochondrial damage [143]. Verteporfin exerts its effects on both YAP-dominant MKN-74 cells and TAZ-dominant MKN-45 cells; however, in MKN-45, the inhibition caused by verteporfin is faster than in that seen in MKN-74 cells [141]. As the YAP and TAZ expression is reduced, verteporfin also decreases the number of downstream genes, such as survivin [141]. In this study, verteporfin induced apoptosis through increasing caspase-3 and caspase-7 by decreasing survivin expression [141]. In NCI-N87 GC cells, verteporfin cytotoxicity was improved when used in combination with paclitaxel, due to increasing apoptotic activity [357]. In sum, verteporfin is useful in suppressing GC cell proliferation and may be a potential agent in GC treatment [143].

#### 5.1.18. Retinoids

The retinoids have been shown to regulate cell growth, differentiation and apoptosis. These agents inhibit carcinogenesis progression [61]. Retinoids affect gene expression through the activation of two receptor families, RARs and RXRs, members of the steroid receptor family. Both RARs and RXRs have α, β and γ isoforms. Heterodimers (RAR-RXR) are formed due to the bond between RARs and RXRs and the retinoid, which bind specific DNA sequences, activating gene transcription to produce drug pharmacological effects. Cellular differentiation and proliferation are affected by the retinoids that target RARs, whereas retinoids that target RXRs induce apoptosis [152].

**Figure 2:**
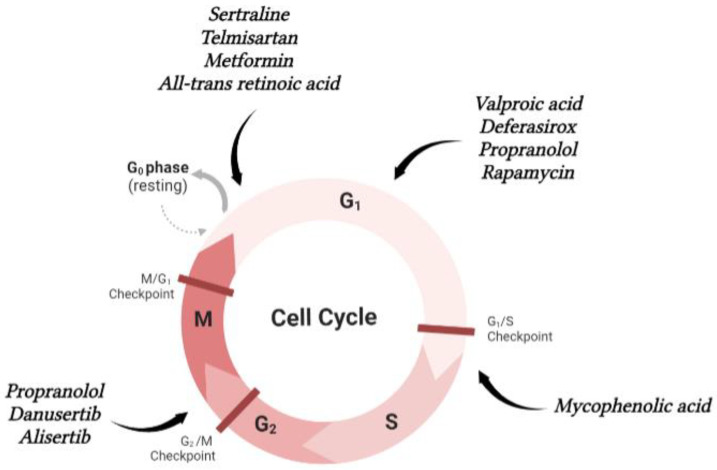
Schematic representation of repurposed drugs for gastric cancer that interfere in cell cycle.

All-trans retinoic acid is a naturally occurring retinoid used in acute promyelocytic leukaemia treatment [61,145,146,147,148]. It also exhibits anticancer activity in glioblastoma [358], ovarian [359] and breast cancers [360]. A study showed that SC-M1 and TSGH9201 GC cells are sensitive to all-trans retinoic acid, whereas AGS cells are partially sensitive and TMK-1 and TMC-1 cells are resistant. The growth suppression is related to cell cycle arrest in the G0/G1 phase [149] (Figure 2).

Additionally, in SC-M1 cells, all-trans retinoic acid increases GC cells’ resistance to lymphokine-activated killer-mediated cytotoxicity through cell cycle regulation [361]. All-trans retinoic acid displays antiproliferative characteristics and induces cell cycle arrest in AGS, MKN-45, MKN-74, NCI-N87, MKN-7 and MKN-28 GC cells by upregulating CDK inhibitors and downregulating activators of the cell cycle. Furthermore, all-trans retinoic acid targets cancer stem cells, downregulating its markers’ expression (CD44 and aldehyde dehydrogenase (ALDH) and stemness genes, including Klf4 and Sox2. In vivo, all-trans retinoic acid is able to inhibit GC progression [150]. Another investigation demonstrated that miR-542-3p coupled with sorafenib/all-trans retinoic acid in a lipid nanoparticle is efficient against GC. In vitro experiments showed that miR-542-3p displays an antiproliferative effect in MGC-803 GC cells and the nanocarrier encapsulation of sorafenib plus all-trans retinoic acid exhibits higher cytotoxicity when compared to individual drugs or combinations, suggesting that the cellular uptake of different formulations affects the therapeutic efficiency [151]. In vivo data indicate that nanoparticle encapsulation of sorafenib and miRNA displays anticancer efficiency, and miRNA coupled with sorafenib/all-trans retinoic acid in a lipid nanocarrier might be a potential therapeutic strategy in GC treatment [151]. Since long noncoding RNAs (LncRNAs) are associated with cancer development, a study found that LncHOXA10 is upregulated in GC, promoting cell proliferation, migration and invasion. On the other hand, RAR-β is downregulated and apoptosis-inhibited. In fact, LncHOXA10 can suppress RAR-β expression and all-trans retinoic acid enables RAR-β expression. Thus, all-trans retinoic acid can reverse the tumourigenic function of LncHOXA10, indicating a new target and drug for GC treatment [362]. 13-cis-retinoic acid is a retinoid that derivates from vitamin A and induces changes in gene expression through binding to specific nuclear receptors, resulting in apoptosis or differentiation of malignant or premalignant cells [153]. It is used for acne vulgaris treatment and also has been proven to be effective in the chemoprevention of aerodigestive malignancies [152,153]. It has antitumour functions in neuroblastoma [363], adult T-cell leukaemia [364] and prostate cancer [365].

A study compared all-trans retinoic acid and 13-cis-retinoic acid action on SC-M1 GC cells in vitro and in vivo. 13-cis-retinoic acid is able to inhibit GC cell growth and also tumour growth. However, the in vitro results showed that all-trans retinoic acid is more effective than 13-cis-retinoic acid, although in vivo 13-cis-retinoic acid displays better results. These data suggest that both all-trans retinoic acid and 13-cis-retinoic acid inhibit SC-M1 GC cell growth [154].

9-Cis-retinoic acid is the endogenous RXR ligand [140]. It is used in cutaneous lesions of Kaposi sarcoma treatment [152]. 9-cis-retinoic acid has shown anticancer potential in cutaneous T-cell lymphoma [366], breast [367,368], prostate [369,370], pancreatic [371,372] and adrenocortical cancers [373] and oral squamous cell carcinoma [374]. 9-cis-retinoic acid displays a cytostatic effect on TMK-1, MKN-1, MKN-28, MKN-45, MKN-74, HSC-39 and KATO-III GC cells synthesizing RARs and RXR-α mRNAs. However, MKN-7 cells do not transcribe the receptors. These data indicate that 9-cis-retinoic acid signal transduction in GC may be associated with RARs and RXR-α synthesis [155]. Retinoic acid treatment continuously produces Waf1/Cip1/Sdi1/p21 with cell cycle arrest in the G0/G1 phase in apoptosis-inducible cells. However, in TMK-1 cells treated with 9-cis-retinoic acid, apoptosis does not occur, and cell cycle arrest in the G0/G1 phase failed in MKN-7-resistant cells and also in TMK-1- and KATO-III-sensitive cells. In GC cells, apoptosis induction by 9-cis-retinoic acid might need constant induction of Waf1/Cip1/Sdi1/p21 [155]. Moreover, 9-cis-retinoic acid decreases the number of CDK7, EGFR and cyclin D1 proteins and reduces phosphorylation of the Rb tumour suppressor gene in TMK-1 cells, but not in MKN-7-resistant cells [155]. In sum, 9-cis-retinoic acid exhibits a cytostatic effect via cell cycle regulation in GC cells, indicating that it might be a potential agent for GC therapy [155].

#### 5.1.19. Selective Oestrogen Receptor Modulator

Bazedoxifene is a selective oestrogen receptor modulator (SERM) used for menopausal symptoms and prophylaxis of postmenopausal osteoporosis [156,157]. This drug exhibits antitumour effects on colon [375], cervical [376], breast [377], ovarian [378] and pancreatic cancers [379], glioblastoma [380,381] and rhabdomyosarcoma [382]. A study of preclinical mouse models for gastrointestinal cancers demonstrated that bazedoxifene displays anticancer activity by reducing the GC tumour burden in gp130^Y757F^ mice, whereas tumours arise through glycoprotein 130 (gp130)/ signal transducer and activator of transcription 3 (STAT3) signalling in response to the Interleukin-6 (IL-6) family cytokine IL-11 [158]. The IL-6 ligands family is characterized by a shared use of the gp130 receptor α-subunit [383]. In cancer, STAT3 activation is associated with poor prognosis, and arises from increased IL-6. In sporadic gastrointestinal cancers, the IL-11/STAT3 signalling axis is a powerful tumour progressor [384]. IL-6 and IL-11 have been identified as potential therapeutic targets for gastrointestinal cancers [383]. Therefore, bazedoxifene may be a potential drug for gastrointestinal cancer treatment, in which IL-11 promotes tumour development [158].

## 6. Conclusions and Future Perspectives

In GC, chemotherapy improves patients’ survival. Combination therapies have also demonstrated survival improvement when compared to a single-agent regimen treatment. In patients with HER-2-positive tumours, targeted therapy protocols using trastuzumab in combination with capecitabine or 5-FU in combination with cisplatin have been proven to be valuable. For those patients with HER-2-negative tumours, protocols with combinations of two and three drugs, including docetaxel, oxaliplatin, irinotecan or 5-FU, consist of effective treatment choices for advanced GC [8]. Drug development approaches demand extreme effort and time to identify molecular mechanisms of action [39]. In order to decrease risks and costs and save time, drug repurposing has become an important part of the drug development procedure [40,41], since it is based on the research of new therapeutic indications of drugs already approved for other pathologies [51], which display a greater potential of being safe in new conditions [40,41]. In GC, several repurposed drugs have been tested in vitro and in vivo, and the majority exhibited positive results, suggesting that they might be effective in this disease treatment. However, further investigation is required to ensure their efficiency and to move on to clinical trials. Definitive information from phase III clinical trials with drug repurposing is still not enough to compare the efficacy of drugs indicated to be first- or second-line therapies, as well as primary or metastatic disease. However, we believe that we can get closer to this reality.

## Figures and Tables

**Table 1:** Repurposed drugs for gastric cancer.

Drug	Drug Class	Clinical Use	GC Cell Lines	Effects In Vitro	Effects In Vivo	References
Fluoxetine	Antidepressant	Depression, obsessive compulsive disorder, panic and anxiety disorders, post-traumatic stress disorder, fibromyalgia and neuropathic pain	AGS	Inhibits cell proliferation and induces apoptosis	-	[42,43,44,45,46,47,48,49]
Sertraline		Major depression, anxiety and panic disorders, obsessive compulsive disorder, post-traumatic stress disorder, perimenopausal vasomotor symptoms, eating disorders, fibromyalgia and neuropathic pain	SGC7901	Induces apoptosis and cell cycle arrest in the G0/G1 phase	-	[42,43,45,46,50]
Paroxetine		Depression, obsessive compulsive disorder, panic and anxiety disorders, post-traumatic stress disorder, fibromyalgia and neuropathic pain	AGS MKN-45	Inhibits cell proliferation and induces apoptosis	-	[42,43,45,46,51]
Valproic acid	Antiepileptic	Mania	AGS SGC-7901 OCUM-2MD3 BGC-823 HGC-27 MKN-28 NCI-N87	Inhibits cell proliferation and migration; induces apoptosis, autophagy and cell cycle arrest in G1 phase	Inhibits cell growth due to autophagy and apoptosis	[52,53,54,55,56,57,58,59]
Lovastatin	Statins	Dyslipidemias	AGS BGC-823 NCI-N87 MKN28 HGT-1	Inhibits cell proliferation and induces apoptosis	HDAC2 suppression	[59,60,61,62,63,64]
Simvastatin			MKN-45 MGC-803 NCI-N87 Hs746T AGS HCG-27 SNU-5 SNU-16 SNU-620 SNU-668 SNU-719 MKN-28 MKN-1	Induces apoptosis; inhibits cell proliferation, migration and invasion	Suppresses tumour growth	[60,61,62,63,65,66,67,68,69,70]
Thioridazine	Antipsychotic	Schizophrenia and bipolar disorder	AGS NCI-N87	Inhibits cell proliferation and induces apoptosis		[54,71,72,73,74,75]
Risperidone		Schizophrenia, acute mania and bipolar disorder	KATO-III	Induces apoptosis		[46,54,71,72,73,76]
Telmisartan	Angiotensin receptor blocker	Hypertension, heart failure and diabetic nephropathy	MKN-74 MKN-1 MKN-45	Inhibits cell proliferation through cell cycle arrest in the G0/G1		[77,78,79]
Candesartan			MKN-45	Suppresses epithelial-to-mesenchymal transition	Inhibits tumour proliferation and fibrosis	[46,77,78,80]
Metformin	Antidiabetic	Type 2 diabetes	MKN1 MKN-45 MKN-74 MKN-28 SGC-7901 BGC-823 AGS HR TSGH HGC27 SGC7901 N87 SNU216 MGC803 KATO-III SNU-1 HGC-27	Inhibits cell proliferation through cell cycle arrest in G0-G1 or G2/M phase; inhibits migration and stemness; induces apoptosis	Suppresses tumour growth and reduces the self-renewal ability of cancer stem cells	[46,60,61,81,82,83,84,85,86,87,88,89,90,91,92,93,94,95,96,97,98,99]
Doxycycline	Antibiotic		AGS MKN-45 KATO III	Inhibits cell proliferation	-	[46,100,101,102]
Tigecycline		Skin and soft-tissue infection, intra-abdominal infections, community-acquired pneumonia	GAM-016 MKN-45	Inhibits cell proliferation and induces autophagy	-	[101,103,104]
Disulfiram	Antialcohol	Alcohol use disorder	MKN-45 SGC 7901 BGC-823 HGC-27 SGC-7901	Inhibits cell proliferation, migration and invasion	Induces autophagy	[46,105,106,107]
Deferasirox	Iron chelator	Iron overload, thalassemia and myelodysplastic syndrome	AGS MKN-28 SNU-484 SNU-638	Inhibits cell growth, induces apoptosis and cell cycle arrest in the G1 phase	-	[108,109,110,111]
Bortezomib	Proteasome inhibitor	Multiple myeloma and mantle cell lymphoma	SNU638 MUGC-3 MKN-28	Inhibits cell proliferation and induces apoptosis	-	[26,46,112,113,114]
Propranolol	Nonselective β-adrenergic Receptor Antagonist	Hypertension, angina pectoris, arrhythmias, migraine, hyperthyroidism, anxiety, tremor, infantile haemangiomas and angiosarcoma	SGC-7901 BGC-823 MKN-45 NUGC3	Inhibits cell proliferation, induces apoptosis and cell cycle arrest in the G1 or G2/M phase	Suppresses cell proliferation and induces apoptosis	[78,115,116,117]
Naftopidil	α-1 Adrenoceptor Blocker	Prostatic hyperplasia	HGC27	Decreases cell viability, induces apoptosis and autophagy	-	[46,118,119]
Naftopidil analogue—HUHS1015		-	MKN-28 MKN-45	Decreases cell viability, induces apoptosis and necrosis	Suppresses tumour growth	[120]
Danusertib	Aurora kinase inhibitor	-	AGS NCI-N78	Arrests cell cycle in the G2/M phase, induces apoptosis and autophagy	-	[121]
Alisertib		-	AGS SNU-1 NCI-N78	Inhibits cell proliferation, induces apoptosis, autophagy and cell cycle arrest in the G2/M phase	Suppresses tumour growth	[122,123]
Abacavir	Nucleoside reverse transcriptase inhibitor	Human immunodeficiency virus (HIV)	AGS	Promotes cellular senescence	-	[46,124,125,126,127]
Propofol	Anaesthetic	Induction/maintenance of anesthesia, sedation	SGC7901 MGC-803 MKN-45 AGS	Suppresses cell proliferation, invasion, migration and induces apoptosis	-	[128,129,130,131,132,133,134]
Levobupivacaine		Pain control during labour, postoperative periods and in patients with chronic pain	HGC27 SGC7901	Inhibits cell proliferation	Inhibits cell proliferation	[135,136]
Mycophenolic acid	Immunosuppressive	-	AGS	Inhibits cell proliferation, induces cell cycle arrest in the G1/S phase	-	[137]
Rapamycin		Post-transplant immunosuppression	TMK-1 MKN-28 MKN-45 MKN-74	Inhibits cell proliferation and induces cell cycle arrest in the G1 phase	-	[112,138]
Verteporfin	Photosensitizer	Choroidal neovascularization	AGS NCI-N87 MKN1 MKN-45 SNU638 MKN-74 KATO-III NUGC-4	Inhibits cell proliferation and induces apoptosis	Reduces tumour growth	[139,140,141,142,143,144]
All-trans retinoic acid	Retinoid	Acute promyelocytic leukaemia	SC-M1 TSGH9201 AGS TMK-1 TMC-1 BGC-823 NCI-N87 SGC7901 MKN-45	Inhibits cell proliferation and induces cell cycle arrest in the G0/G1 phase	Inhibits tumour progression	[61,145,146,147,148,149,150,151]
13-cis-retinoic acid		Acne vulgaris	SC-M1	Inhibits cell growth	Suppresses tumour growth	[152,153,154]
9-cis-retinoic acid		Cutaneous lesions of Kaposi sarcoma	TMK-1 MKN-1 MKN-28 MKN-45 MKN-74 HSC-39 KATO-III MKN-7		-	[152,155]
Bazedoxifene	Selective oestrogen receptor modulator	Menopausal symptoms and prophylaxis of postmenopausal osteoporosis	-	-	Reduces tumour burden	[156,157,158]

## Data Availability

Not applicable.
